# Supplementary figures and images for: Multiplex PCR assay to identify clinically important Aeromonas species
Source: Microbiol Spectr. 2025 Apr 9;13(5):e03331-24. doi: 10.1128/spectrum.03331-24 (PMC12054095; doi:10.1128/spectrum.03331-24)

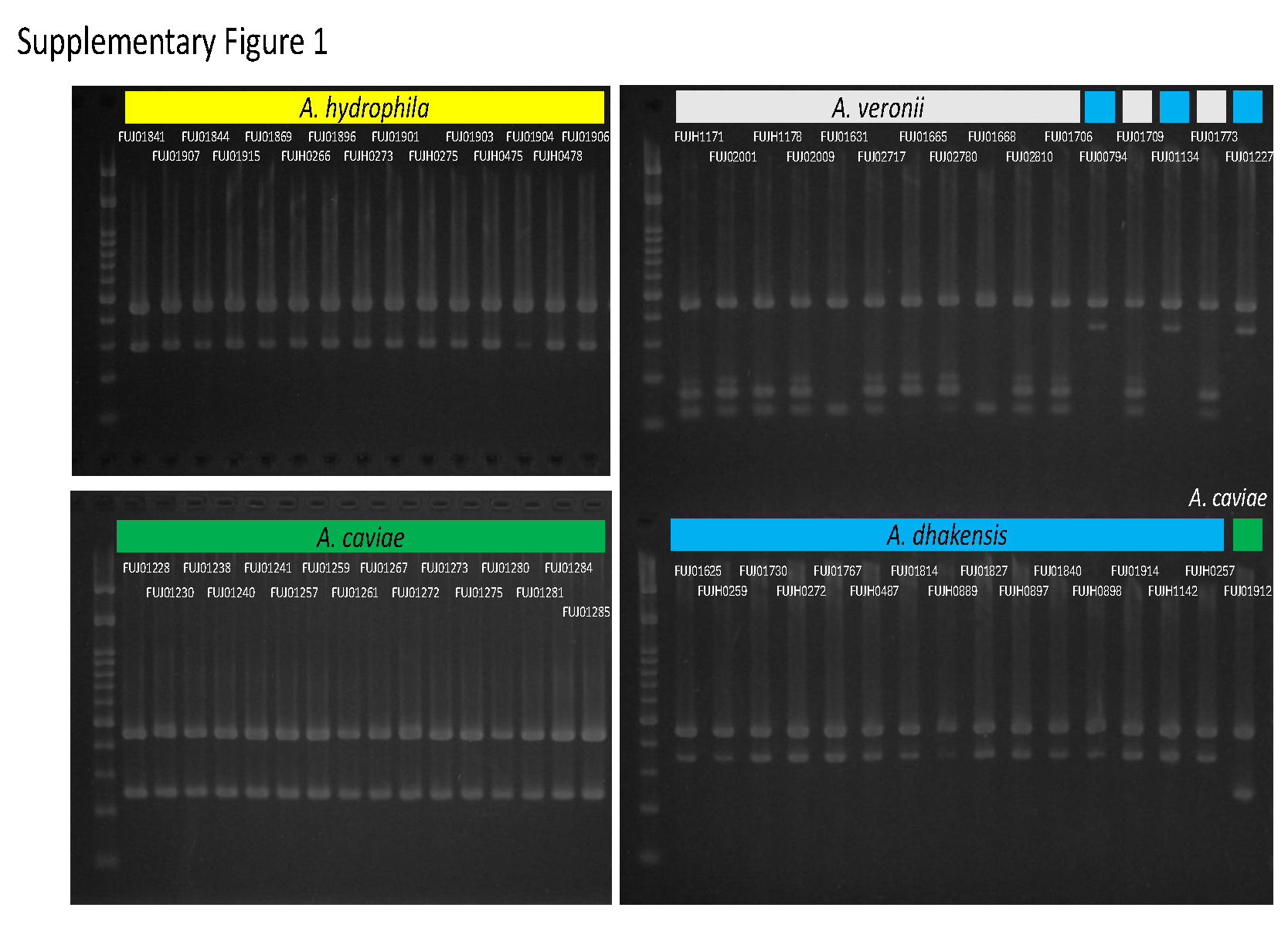

Supplement: Fig. S1 — Gel image of multiplex PCR products from selected clinical strains. [file spectrum.03331-24-s0001.tif]
