# Supplementary material for: Multiplex PCR assay to identify clinically important Aeromonas species
Source: Microbiol Spectr. 2025 Apr 9;13(5):e03331-24. doi: 10.1128/spectrum.03331-24 (PMC12054095; doi:10.1128/spectrum.03331-24)
Supplement: Supplemental legends — Legends for supplemental figures and tables. [file spectrum.03331-24-s0002.docx]

***Supplementary Appendix***

**Supplementary Table 1**. Reference genomes representing 31 *Aeromonas* species.

**Supplementary Table 2.** List of NCBI *Aeromonas* genomes.

**Supplementary Table 3.** Presence (=1) or absence (=0) of candidate ORFs in NCBI *Aeromonas* genomes. Candidate ORF regions with ≥80% nucleotide sequence identity and ≥80% coverage in the genome of each strain were considered present (=1) and displayed as colored blocks accordingly.

**Supplementary Table 4.** List of clinical *Aeromonas* strains.

**Supplementary Figure 1**. Gel image of multiplex PCR products from selected clinical strains. The leftmost lane contains a 100-bp DNA marker. Lanes beneath the yellow box correspond to PCR products from clinical strains of *A. hydrophila*, those beneath the green tab to *A. caviae*, those beneath the blue tab to *A. dhakensis*, and those beneath the gray box to *A. veronii*.
