# Supplementary material for: Multiplex PCR assay to identify clinically important Aeromonas species
Source: Microbiol Spectr. 2025 Apr 9;13(5):e03331-24. doi: 10.1128/spectrum.03331-24 (PMC12054095; doi:10.1128/spectrum.03331-24)
Supplement: Table S1 — Reference genomes representing 31 Aeromonas species. [file spectrum.03331-24-s0003.docx]

**Supplementary Table 1**. Reference genomes representing 31 species of the genus A*eromonas*

| *Aeromonas* species | Strain | Type strain | NCBI RefSeq  Assembly Accession | Level | Size | BioProject | BioSample | Submission Date |
| --- | --- | --- | --- | --- | --- | --- | --- | --- |
| *A. allosaccharophila* | DSM 24388 | yes | GCF_016026615.1 | Complete | 4630407 | PRJNA231221 | SAMN13450463 | 12/14/2020 |
| *A. aquatica* | AE235 | yes | GCF_000764655.1 | Contig | 4582304 | PRJNA260478 | SAMN03023875 | 10/8/2014 |
| *A. australiensis* | CECT 8023 | yes | GCF_000819725.1 | Contig | 4110035 | PRJEB7021 | SAMEA2752426 | 12/1/2014 |
| *A. bestiarum* | GA97-22 | no | GCF_002906925.1 | Contig | 4714837 | PRJNA431444 | SAMN08391293 | 1/30/2018 |
| *A. bivalvium* | ZJ19-2 | no | GCF_003265465.1 | Scaffold | 4256224 | PRJNA408193 | SAMN07680236 | 6/26/2018 |
| *A. caviae* | NCTC 12244 | yes | GCF_900476005.1 | Complete | 4586140 | PRJEB6403 | SAMEA4475690 | 6/17/2018 |
| *A. dhakensis* | KN-Mc-6U21 | no | GCF_002285935.1 | Chromosome | 4868053 | PRJNA400818 | SAMN07577462 | 9/5/2017 |
| *A. diversa* | CECT 4254 | yes | GCF_000819805.1 | Scaffold | 4062507 | PRJEB7026 | SAMEA2752422 | 12/1/2014 |
| *A. encheleia* | NCTC12917 | yes | GCF_900637545.1 | Complete | 4542521 | PRJEB6403 | SAMEA4475689 | 12/20/2018 |
| *A. enteropelogenes* | DSM 9382 | yes | GCF_020341435.1 | Chromosome | 4354161 | PRJNA231221 | SAMN21402704 | 10/7/2021 |
| *A. eucrenophila* | CECT 4224 | yes | GCF_000819865.1 | Scaffold | 4540059 | PRJEB7029 | SAMEA2752419 | 12/1/2014 |
| *A. finlandensis* | 4287D | yes | GCF_000764645.1 | Contig | 4717507 | PRJNA260478 | SAMN03023686 | 10/8/2014 |
| *A. fluvialis* | LMG 24681 | yes | GCF_000819885.1 | Scaffold | 3904228 | PRJEB7030 | SAMEA2752418 | 12/1/2014 |
| *A. hydrophila* | ATCC 7966 | yes | GCF_000014805.1 | Complete | 4744448 | PRJNA16697 | SAMN02604052 | 11/6/2006 |
| *A. jandaei* | DSM 7311 | yes | GCF_016127195.1 | Complete | 4551079 | PRJNA231221 | SAMN16357155 | 12/20/2020 |
| *A. lacus* | AE122 | yes | GCF_000764665.1 | Contig | 4394373 | PRJNA260478 | SAMN03023876 | 10/8/2014 |
| *A. lusitana* | MDC 2473 | yes | GCF_002812985.1 | Scaffold | 4537799 | PRJNA417247 | SAMN07981238 | 11/16/2017 |
| *A. media* | TR3_1 | no | GCF_020423125.1 | Complete | 4531033 | PRJNA730636 | SAMN19237318 | 10/12/2021 |
| *A. molluscorum* | 848 | yes | GCF_000388115.1 | Contig | 4236171 | PRJNA183610 | SAMN02471397 | 5/3/2013 |
| *A. piscicola* | LMG 24783 | yes | GCF_000820005.1 | Scaffold | 5177970 | PRJEB7033 | SAMEA2752415 | 12/1/2014 |
| *A. popoffii* | CIP 105493 | yes | GCF_000820025.1 | Scaffold | 4762473 | PRJEB7034 | SAMEA2752414 | 12/1/2014 |
| *A. rivipollensis* | KN-Mc-11N1 | no | GCF_003015165.1 | Complete | 4508901 | PRJNA438570 | SAMN08721782 | 3/26/2018 |
| *A. rivuli* | 20-VB00005 | no | GCF_020149575.1 | Complete | 4357928 | PRJNA747139 | SAMN20256631 | 9/27/2021 |
| *A. salmonicida* | AS3 | no | GCF_028355655.1 | Complete | 4954811 | PRJNA896320 | SAMN31541908 | 1/31/2023 |
| *A. sanarellii* | LMG 24682 | yes | GCF_000820085.1 | Scaffold | 4186903 | PRJEB7037 | SAMEA2752411 | 12/1/2014 |
| *A. schubertii* | ATCC 43700 | yes | GCF_001481395.1 | Scaffold | 4140228 | PRJNA304368 | SAMN04299626 | 12/22/2015 |
| *A. simiae* | A6 | no | GCF_014892695.1 | Complete | 3974097 | PRJNA543361 | SAMN11664857 | 10/9/2019 |
| *A. sobria* | CECT 4245 | yes | GCF_000820145.1 | Scaffold | 4683669 | PRJEB7040 | SAMEA2752408 | 12/1/2014 |
| *A. taiwanensis* | LMG 24683 | yes | GCF_000820165.1 | Scaffold | 4250891 | PRJEB7041 | SAMEA2752407 | 12/1/2014 |
| *A. tecta* | CECT 7082 | yes | GCF_000820185.1 | Scaffold | 4755221 | PRJEB7042 | SAMEA2752406 | 1/30/2015 |
| *A. veronii* | FDAARGOS_632 | no | GCF_008693705.1 | Complete | 4561870 | PRJNA231221 | SAMN11056347 | 9/25/2019 |
